# Identification of hub genes related to prognosis in glioma

**DOI:** 10.1042/BSR20193377

**Published:** 2020-05-27

**Authors:** Delong Zhang, Jinxia Zhao, Chengzheng Han, Xiaocen Liu, Jun Liu, Hui Yang

**Affiliations:** 1 Key Laboratory of Non-coding RNA Transformation Research of Anhui Higher Education Institution (Wannan Medical College), Wuhu 241001, Anhui, People’s Republic of China; 2 Central Laboratory, The First Affiliated Hospital of Wannan Medical College, Wuhu 241001, Anhui, People’s Republic of China; 3 Non-coding RNA Research Center of Wannan Medical College, Wuhu 241001, Anhui, People’s Republic of China; 4 Department of Anesthesiology, The First Affiliated Hospital of Wannan Medical College, Wuhu 241001, Anhui, People’s Republic of China; 5 Clinic of Integrated Traditional Chinese and Western Medicine, The First Affiliated Hospital of Wannan Medical College, Wuhu 241001, Anhui, People’s Republic of China; 6 Department of Nuclear Medicine, The First Affiliated Hospital of Wannan Medical College, Wuhu 241001 Anhui, People’s Republic of China; 7 Department of Neurosurgery, The First Affiliated Hospital of Wannan Medical College, Wuhu 241001, Anhui, People’s Republic of China

**Keywords:** bioinformatics, gene, glioma

## Abstract

Glioma, a common malignant tumor of the central nervous system, has high invasiveness. The objective of the present study was to identify genes playing an important role in the development of glioma and to reveal their potential research value. Conjoint analysis on the GSE16011 dataset in the Gene Expression Omnibus (GEO) database and the ‘Messenger RNA Expression Microarray of Diffuse Gliomas and Controls’ dataset and ‘RNA sequencing of Diffuse Gliomas’ dataset in the Chinese Glioma Genome Atlas (CGGA) database is carried out in the study. The weighted correlation network analysis (WGCNA) was used to carry out co-expression analysis on the GSE16011.

Finally, 24 genes significantly related to grade and prognosis were obtained. In addition, there is no report about CACNG2, JPH3, TUBB6 (tubulin β 6 class V), NRSN1, FAM19A2, NALCN, CDH18, GNAL on glioma.

## Background

Malignant gliomas are the most common primary brain tumors in adults. Usually, low-grade gliomas (WHO I/II) are not diagnosed in time while high-grade gliomas (WHO III/IV) have a poor prognosis due to high degree of malignancy. The median survival period of gliomas is only 12–15 months [1,2]. Besides, the progression of the tumors is involved with many genes. Glioma accounts for approximately 30% of the central nervous system tumors, for 80% of brain malignancies [3]. Gliomas is divided into four grades – grade I to grade IV, according to WHO classification [4]. The higher the gliomas grade is, the higher the invasive ability and the recurrence rate are, helping explain that studying the pathogenesis of glioma is helpful to the identification of potential drug targets.

Gene chip and RNA sequencing are technologies used to detect gene expression, the application history of which has exceeded 10 years. These technologies can rapidly compare expression levels of a large number of genes in different samples, making them suitable for gene screening [5]. With the development of global genome research and promotion for sharing scientific research data, many public databases including miRNA, lncRNA, messenger RNA (mRNA) and circRNA can be found on the internet, providing a more credible basis for global researchers to start new projects. It is possible to identify credible differential expressions based on open databases involved with a large number of samples. In the present study, we try to find genes in glioma that are significantly associated with WHO grading and prognosis, thereby making it necessary to select datasets with large sample size capable of assisting us to draw up credible conclusions and complete clinical information.

Weighted gene co-expression network analysis (WGCNA) serves as a tool for analysis of gene co-expression, initially, whose mathematical principle was proposed by Zhang and Horvath [6] in 2005. Langfelder and Horvath [7] implemented this algorithm in R analytic environment, named as WGCNA package, in 2008 [8]. Recent decades have witnessed a more extensive application of WGCNA package gradually in bioinformatics due to the fact that the algorithm is more reliable than the conventional linear regression analysis and also applicable to the analysis of large sample size. Even more, some co-expression phenomena obtained through this algorithm can even predict binding sites in STRING database (https://www.string-db.org). In the present study, the WGCNA algorithm was used to identify the key genes relate to WHO grading, followed by the screening out of biomarker candidates clearly differentially expressed and significantly related to survival in these genes. The purpose of introducing WGCNA is to obtain a weighted gene co-expression network, further broadening the thoughts of experiments.

At last, 24 genes were identified: VIM, CLIC1, TUBB6 (tubulin β 6 class V), SERPINH1, ANXA2, COL1A2, COL4A1, COL4A2, TIMP1, ANXA1, COL1A1, COL3A1, TACC3, KIF20A, FN1, CACNG2, KCNB1, CDH18, GNAL, NRSN1, SH3GL2, JPH3, NALCN, FAM19A2. Some genes were proven to be related to tumor functions while the others (among these 24 genes) such as FN1, COL4A2, COL4A1, COL1A2, were consistent with conclusions of key genes drawn up based on other datasets by Hao et al. [9]. The frequent report of these two genes – VIM and CLIC1 has proved high credibility of our conclusions.

## Materials and methods

### Gene expression profile

Gene Expression Omnibus (GEO) dataset GSE16011 was downloaded using the GEO query package in R [10]. RNA sequencing of Diffuse Gliomas (abbreviated as ‘CGGA RNAseq FPKM data’) [11] and Messenger RNA Expression Microarray of Diffuse Gliomas and Controls (abbreviated as ‘CGGA mRNA microarray data’) [12] were downloaded from Chinese Glioma Genome Atlas (CGGA) website (http://www.cgga.org.cn/). GSE16011 included 8 normal brain tissue samples, 8 samples of WHO grade I, 24 samples of grade II, 85 samples of grade III and 159 samples of grade IV, i.e. 284 samples in total. However, survival data of patients were not included in this dataset. CGGA RNAseq FPKM data included 109 samples of WHO grade II, 72 samples of grade III and 144 samples of grade IV, i.e. 325 samples in total, along with data over survival period. CGGA mRNA microarray data included 122 samples of WHO grade II, 51 samples of grade III and 128 samples of grade IV, i.e. 301 samples in total, along with data over survival period. GSE16011 and CGGA mRNA microarray data were subjected to quantile normalization before differential analysis.

### Online tools

DAVID website (https://david.ncifcrf.gov) was for GO or KEGG enrichment analysis.

### WGCNA

WGCNA was applied to identify WHO-grade-related networks. In this section, the steps of WGCNA and how to identify significant modules associated with glioma grades are explained, along with description of how to identify the most representative genes in the module. WGCNA is responsible for ‘Topological Overlap Matrix’ (TOM), in which, greater the TOM value is, the stronger co-expression of the two genes is.

First, it is needed for WGCNA to calculate the correlation coefficient of two genes’ expression profiles, followed by the calculation of two weighted co-expression networks – unsigned and signed by WGCNA. According to different choices, WGCNA has two formulas for calculating correlation coefficient: $S_{ij}^{unsigned}\;=\;abs(cor(x_{i},x_{j})),\;S_{ij}^{signed}\;=\;cor(x_{i}x_{j},)/2+0.5$.

And then, the soft thresholding is used to carry out the power to S_ij_, and the whole correlation coefficient matrix will more conform to scale-free standard after soft thresholding transformation. Finally, the TOM value, the weight value between nodes of network, will be calculated by WGCNA according to this correlation coefficient matrix.

Unsigned weighted co-expression network generated under soft threshold was set as 9. After co-expression modules were simulated, top four modules significantly related to WHO grading were identified, top 20% representative genes in which were saved as result A.

### Identify the differential expression gene

Differential expression analysis was performed using the limma package in R language. In the present study, samples from GSE16011 data were divided into three groups: normal group, low-grade group (WHO I/II) and high-grade group (WHO III/IV). Genes with the same differential expression trend in DEGs (tumor vs normal) and DEGs (high group vs low group) were examined by chi-square tests, followed by the selection of genes with chi-square significance <0.001.

The samples of CGGA RNAseq FPKM data were divided into three groups: WHO grade II, grade III and grade IV, along with the selection of genes with the consistent trend in DEGs (WHO III vs II) and DEGs (WHO IV vs III).

Selected DEGs identified from GSE16011 ultimately intersect with selected DEGs identified from CGGA RNAseq FPKM data, in which, genes with consistent trend were saved as result D. The CGGA RNAseq FPKM data was only subjected to differential expression analysis without chi-square test to avoid overmining. In addition, differential expression analysis on CGGA mRNA microarray data in order to avoid overmining was not performed.

### Survival analysis

Survival analysis was performed using the ‘survival’ package in R. But survival package was only able to process one gene at a time. To solve this problem, we wrote the R code to process the entire expression matrix. Besides, expression values of each gene and sample prognosis information were combined together and saved into an independent csv file which will be read one by one by a loop invoking ‘survival’ package. In survival analysis, 30% of the low-expression samples were included in the low-expression group while 70% of the high-expression samples were included in the high-expression group, followed by the calculation of the mortality of the samples in each group and relative risk rate (RR value) calculated by mortality rate of the high-expression group divided by mortality rate of the low-expression group were used to judge the relationship between expression levels and death. High-expression levels could be considered to promote death events if RR > 1 while low-expression levels could be considered to promote death events if 0 < RR < 1. A large sample size will be needed for this simple algorithm if it wants to reflect the prognostic trend correctly. The sample size of CGGA database used in the present study is enough.

We carried out survival analysis on CGGA mRNA microarray data and CGGA RNAseq FPKM data. Genes with log-rank *P*-value < 0.001 in both datasets were examined by chi-square tests, and then genes with chi-square significance <0.001 were saved as result C.

### Patients

In total, 10 samples of normal tissue, 21 WHO Ⅱ samples, 22 WHO III samples, 20 WHO IV samples were collected. These 10 normal tissue samples and 63 glioma samples were collected from the Department of Neurosurgery of Yijishan Hospital of Wannan Medical College. The 10 normal samples were extracted from damaged brain tissue of patients with brain trauma. The diagnosis of glioma was performed following the WHO criteria [4]. The inclusion criteria were as follows: patients in the age range of 40–80 years with no serious chronic disease, no history of other cancers and no history of exposure to pollutants (namely, radioactive pollution and carcinogenic chemical pollution). Glioma tissues were stored in liquid nitrogen.

### RT-qPCR

Total RNA was extracted from tissues or cells using TRIzol and stored at −80°C. LncRNA and mRNA were reverse transcribed into complementary DNA with the RevertAid First Strand cDNA Synthesis Kit (#K1622; Thermo Fisher Scientific, Inc., Waltham, MA, U.S.A.) on a GT9612 Gradient Thermal Cycler. RT-qPCR was performed with the QuantiNova™ SYBR® Green PCR kit on a QuantStudio 3 Real-Time PCR System. GAPDH served as an internal standard.

The experimental protocol and reaction conditions complied with the manufacturer’s instructions. The sequences of the primers: GAPDH forward primer, 5′-GCCTGCTTCACCACCTTCT-3′, and GAPDH reverse primer, 5′-GAACGGGAAGCTCACTGG-3′; CACNG2, forward primer, 5′-ACGAAGACCGAAGGTTGCAT-3′, reverse primer, 5′-ATCACACGGGAAGAGGCTTG-3′, FAM19A2 (TAFA2), forward primer, 5′-GCAGGACCGTTGTTTGCTC-3′, reverse primer, 5′-CCCGTATAGTGCAGCGAGAA-3′, GNAL, forward primer, 5′-ACAACTGCCTGGTACTTTGTCA-3′, reverse primer, 5′-AGGGACTCTCTCAGCCTGTT-3′, JPH3, forward primer, 5′-TACGGGACCGAGACCTACTC-3′, reverse primer, 5′- GGTGAAAACGATGCAGCCAC-3′, NALCN, forward primer, 5′-AGTGGGGATGACCTTCTGGATA-3′, reverse primer, 5′-TTATGCCTTTCTGTGGCAGGAT-3′, NRSN1, forward primer, 5′-TGCTTAAGATGGGTGCTTCCTT-3′, reverse primer, 5′-CAGCACCACGCAGAAAACATTA-3′, TUBB6, forward primer, 5′-CGTCCGCAGAGCCAGTTC-3′, reverse primer, 5′-CTTCCCAAAACTTGGTGCCG-3′.

### Statistical analysis

Most statistical analyses were performed in R (version 3.5.0) [13]. The identification standard of differentially expressed genes in the present paper was *P*<0.001, |log2FC| > 1.

Chi-square test was performed for result B: in one dimension, expression value intervals of a gene were divided into three equal parts, which were low expression, medium expression and high expression, to split the samples into the corresponding group. In the other dimension, the samples were divided into high-grade group and low-grade group, which was considered statistically significant when *P*<0.001.

Samples were divided into high-expression group (70% of highest expression samples) and low-expression group (30% of lowest expression samples) for the survival analysis of result D. Likelihood test was used for statistical test, which was considered statistically significant when *P*<0.001.

Chi-square test performed for result D: in one dimension, the samples were divided into high-expression group and low-expression group; in the other dimension, the samples were divided into death group and alive group, which was considered statistically significant when *P*<0.001.

A ‘*’ in statistical graph indicates *P*<0.05, ‘**’ indicates *P*<0.01 and ‘***’ indicates *P*<0.001.

## Results

### WGCNA (result A)

**Figure 1:**
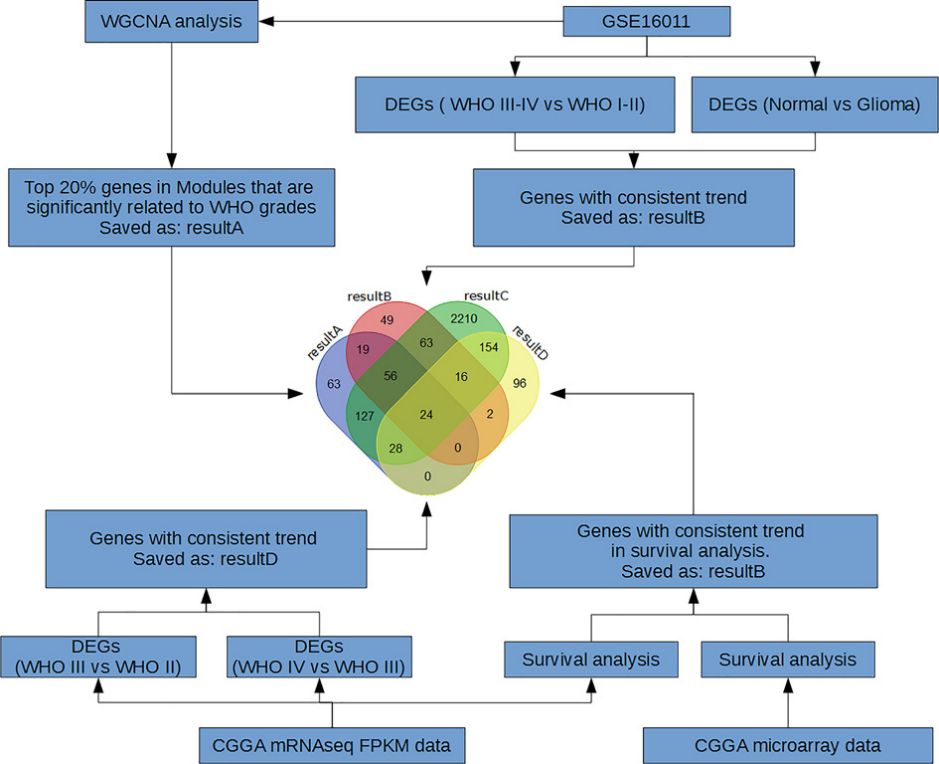
Flow chart for bioinformatics analysis

**Figure 2:**
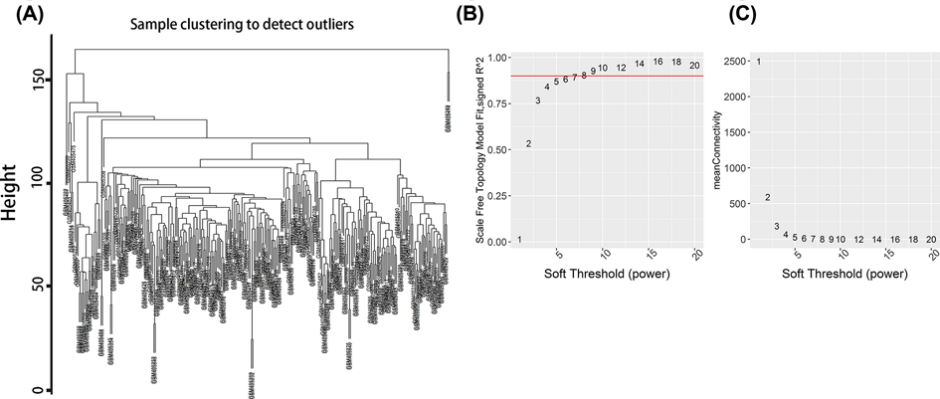
Sample clustering of WGNCA and scale-free topology model fit (**A**) Hierarchical sample clustering. Outlier samples are eliminated according to sample dendrogram. (**B**) Horizontal axis indicates power parameter; vertical axis indicates scale-free fit goodness. Red line indicates threshold 0.9 of fit goodness. Soft threshold with fit goodness being more than 0.9 is required to be selected. (**C**) Mean connectivity of network. Soft threshold with larger connectivity is required to be selected, helping explain the selection of 0.9 as the value of soft threshold.

The unsigned co-expressed network was used in the present study (Figure 1). First, built-in functions of WGCNA package were called for data cleaning, to eliminate the genes or samples whose average expression value was close to 0. All genes and samples in this dataset were not excluded at this step, followed by the cluster analysis on samples to eliminate outlier samples (Figure 2A).

Four samples (GSM405232, GSM405389, GSM405414, GSM405467) were excluded by thresholding height at 150 in the previous cluster diagram.

An appropriate soft threshold should be chosen by scale-free topology model fit. The soft threshold with R^2^ (goodness of fit) >0.9 and largest mean connectivity would be applied to WGCNA. In this research, soft threshold was 9 (Figure 2B,C).

**Figure 3:**
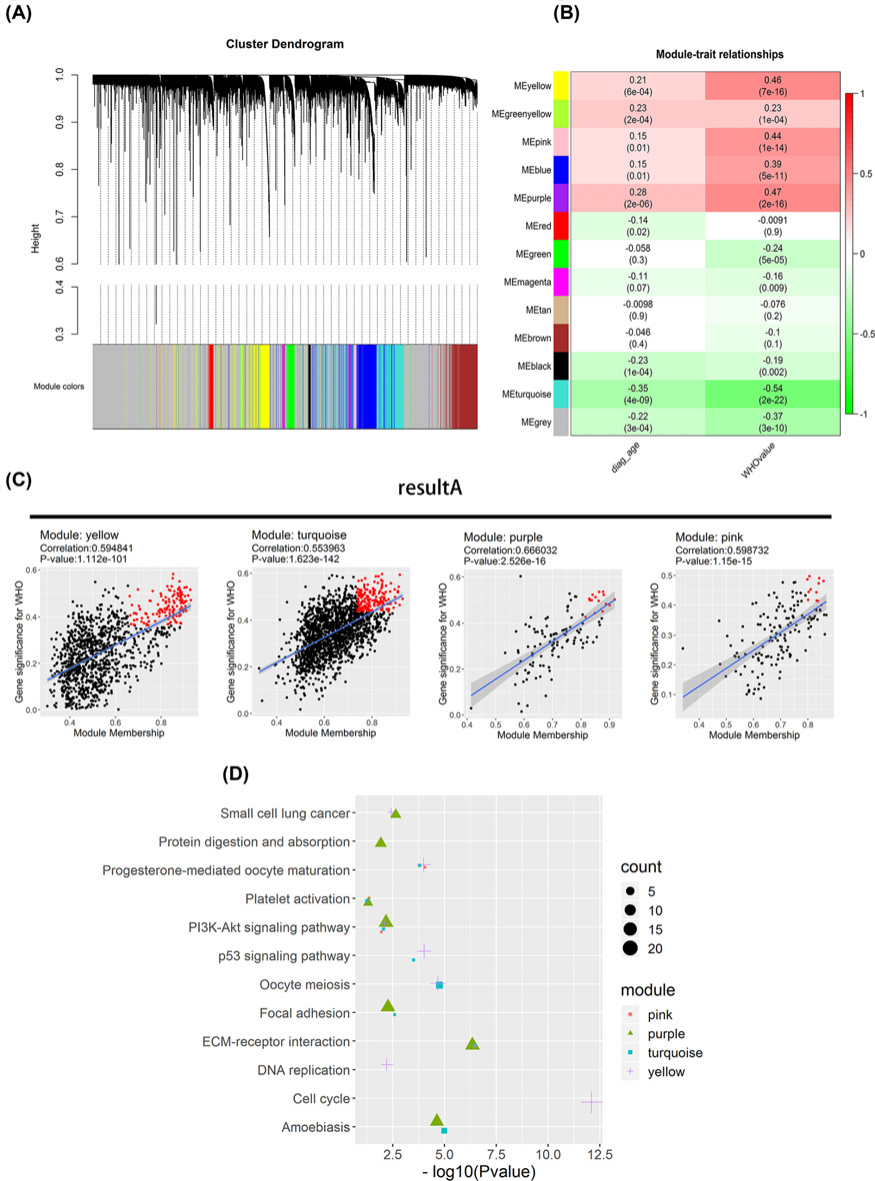
Identification of top 20% of hub genes for WGCNA (**A**) Distance matrix is made according to 1-TOM value for clustering and module division. (**B**) Pearson correlation coefficient between module eigenvalues and clinical indicators. ‘Diag_age’ indicates diagnostic age; ‘WHO value’ indicates WHO grading. Color of heat map indicates correlation coefficient. Numerical value in the bracket indicates *P*-value of correlation coefficient. It is shown in the figure that the module obviously related to WHO grading is yellow, purple, pink, turquoise. (**C**) MM vs GS plot was drawn, with the most representative top 20% genes (result A) in yellow, purple, pink, turquoise modules shown as red dots. (**D**) The KEGG pathway enrichment of the interesting modules.

Thirteen modules were simulated (Figure 3A), and module eigenvectors (MEs) capable of reflecting the overall expression trend of genes in each module, of these modules were calculated. ME and clinical indicators were subject to Pearson correlation analysis to estimate the correlation between each module and clinical indicators.

Based on this result (Figure 3B), we found that no module had significant correlation with diagnostic age and that several modules were significantly correlated with WHO grading. Therefore, we chose the four modules (yellow, purple, pink and turquoise) most relevant to WHO grading for further analysis.

To identify the first 20% genes with the most representative nature in each module, the gene ‘Module Membership’ which could reflect the representativeness of the gene in each module was calculated. The value of gene ‘Module Membership’ referred to the Pearson correlation coefficient between the expression profile of each gene and ME while the gene ‘Trait Significance’ referred to the Pearson correlation coefficient between the expression profile of each gene and clinical traits. The relationship between the level of gene expression and clinical traits could be reflected by ‘Trait Significance’. The MM vs GS figure could reflect the most representative genes in the modules (Figure 3C). Twenty percent of the largest genes in x-axis and 20% of the largest genes in y-axis were subject to intersection. The number of genes in intersected areas was actually less than 20% of the total number of genes in the modules. The most representative genes in these four modules selected by us were regarded as Result A (Figure 3C, additionally shown in Supplementary ResultA.csv) and were subject to KEGG pathway enrichment, from which it was found that they were significantly enriched in cell cycle, ECM–receptor interaction, p53 signaling pathway, PI3K-Akt signaling pathway and small cell lung cancer with the nature as a pathway, which were all clearly associated with tumor function (Figure 3D).

### Differential expression analysis (result B, result D)

**Figure 4:**
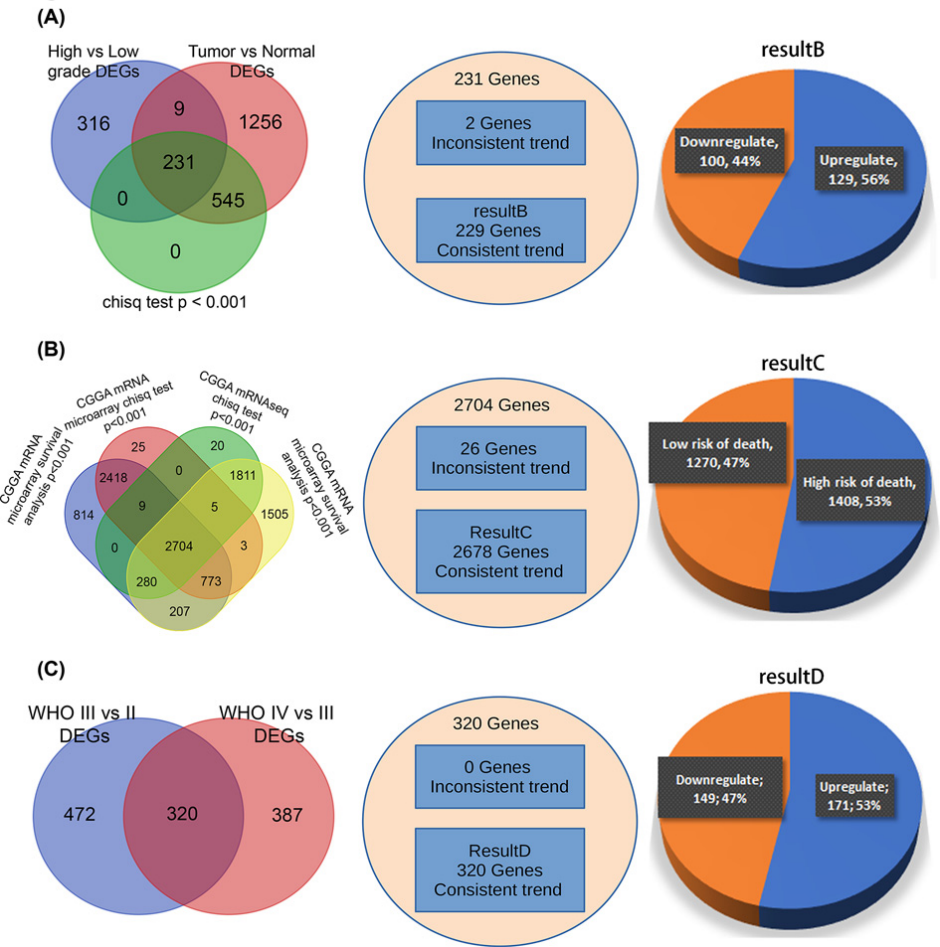
Briefing on result B, result C, result D (**A**) DEGs (high-expression group vs low-expression group), DEGs (tumor vs normal). Chi-square test was conducted for expressing quantity. Three results intersected with each other, obtaining 231 genes. However, only 229 genes have consistent trends in two differential expression analysis. (**B**) Results of survival analysis for CGGA mRNA microarray data and CGGA RNAseq data. Chi-square test was conducted for expressing quantity. It is found that 2707 genes in total were significantly related to prognosis. However, 26 genes are inconsistent trends in two datasets. A total of 2681 genes were finally obtained. (**C**) Differential expression analysis was conducted for CGGA mRNAseq FPKM data. DEGs (WHO III vs II) intersected with DEGs (WHO IV vs III), obtaining 320 genes, all of which have consistent trends in two differential expression analysis.

GSE16011 and CGGA RNAseq FPKM data were subject to differential expression analysis, the former was identified to have 231 differential genes in total. However, genes with consistent trends were saved as result B (Figure 4A, additionally shown in Supplementary ResultB.csv), including 129 significantly up-regulated genes and 100 down-regulated genes. In total of 320 differential genes were identified in CGGA RNAseq FPKM data, which, thereby, were considered as result D (Figure 4C, additionally shown in Supplementary ResultD.csv), including 171 up-regulated and 149 down-regulated genes.

### Survival analysis (result C)

Survival analysis was performed on all genes in CGGA RNAseq FPKM data and CGGA mRNA microarray data. Genes with likelihood test *P*<0.001 and consistent trends in two datasets were screened, with 2682 genes in total regarded as result C (Figure 4B, additionally shown in Supplementary ResultC.csv).

### Unreported key genes

**Figure 5:**
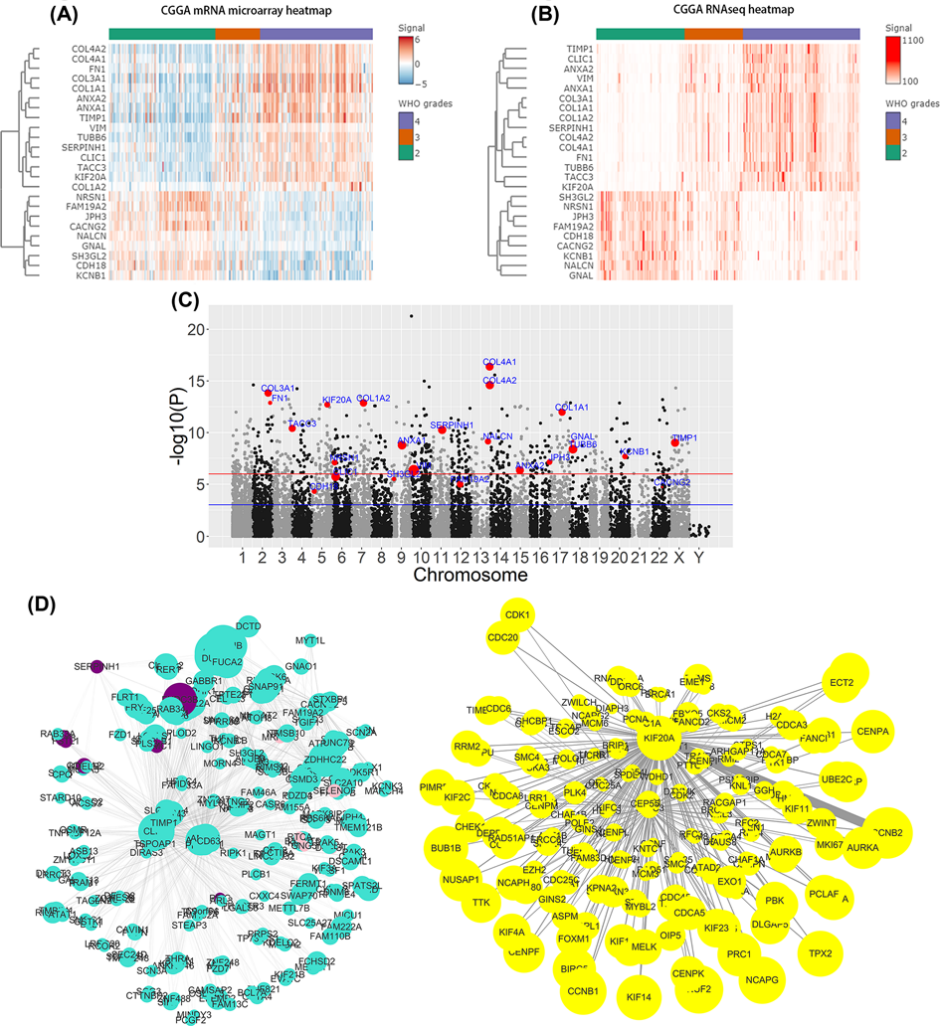
The analyses of 24 key genes (**A**) Heat map indicates the relation of expression of 24 key genes with WHO grades in CGGA mRNA microarray data. (**B**) Heat map indicates the relation of expression of 24 key genes with WHO grades in CGGA mRNAseq FPKM data. (**C**) Manhattan plots presents a visualization analysis of chromosome locus via mapping the initiation site information. This figure was built by plotting −log10 (*P* of DEGs between High and Low in GSE16011) on the y-axis and the chromosome locus on the x-axis, with the highlight of 24 key genes in red. The size of those highlighted points indicates the negative log *P*-value of survival analysis. (**D**) Genes belonging to four yellow, purple, pink, turquoise modules were used to construct the complete co-expressed network obtained through WGCNA. The connection with top 1% connection weights was kept, leading to the disconnection of lots of weak connections in the network. Twenty-four key genes were considered as starting points of searching adjacent nodes and building co-expressed network. Thirty percent of genes with the highest connectivity are shown in the figure (70% of nodes are concealed. Effective information fails to be provided due to excessive nodes and lots of connections). Nodes size in the figure indicates connectivity of nodes. Color indicates module of genes and line width indicates connection weight.

Result A, result B and result D intersected with each other so as to obtain 24 genes in total, including TUBB6, COL4A2, TIMP1, COL4A1, CLIC1, COL1A1, TACC3, COL3A1, VIM, SERPINH1, NALCN, COL1A2, JPH3, FAM19A2, KCNB1, GNAL, KIF20A, ANXA2, FN1, ANXA1, CACNG2, NRSN1, CDH18, SH3GL2. Coincidentally, all these 24 genes belong to result C, indicating that their relation with survival is significant (Table 1, additionally shown in Supplementary Report.csv). CGGA mRNA microarray data and CGGA mRNAseq FPKM data were used to draw heat map for these key genes. Although we only performed a difference analysis on GSE16011 and CGGA mRNAseq FPKM data, the 24 key genes also showed a consistent trend in CGGA mRNA microarray data (Figure 5A,B). Additionally, genetic mapping information showed that DEGs (high vs low in GSE16011) were associated with the negative log of the corresponding *P*-value between 1.0 × 10^−3^ and 0. Four key genes were between 1.0 × 10^−3^ and 1.0 × 10^−6^, 20 key genes at more than 1.0 × 10^−6^ (Figure 5C). Weighted gene co-expressed network of key genes was extracted from the entire weighted gene co-expressed network (Figure 5D).

**Table 1:** Report for 24 key genes (19 May 2018)

Symbol	Affy_ID	Connectivity*	logFC G3 vs G2*	logFC G4 vs G3*	Likelihood test *P*-val*	RR*	Reported in glioma
KIF20A	10112_at	48.96	1.58^†^	1.22^†^	7.56E-15	2.69	Yes
CLIC1	1192_at	39.53	1.12^†^	1.19^†^	1.04E-20	3.80	Yes
ANXA2	302_at	33.12	1.63^†^	1.34^†^	1.43E-19	3.63	Yes
ANXA1	301_at	32.26	1.94^†^	1.27^†^	1.03E-20	3.30	Yes
TIMP1	7076_at	26.38	2.16^†^	2.17^†^	6.81E-20	3.30	Yes
CACNG2	10369_at	24.99	−2.01^†^	−1.77^†^	1.21E-11	0.56	NO
JPH3	57338_at	24.60	−1.23^†^	−1.78^†^	1.90E-14	0.50	NO
VIM	7431_at	22.27	1.31^†^	1.22^†^	2.52E-28	7.29	Yes
TACC3	10460_at	21.63	1.07^†^	1.08^†^	3.10E-17	2.77	Yes
SERPINH1	871_at	14.72	1.32^†^	1.10^†^	2.08E-20	3.74	Yes
KCNB1	3745_at	14.40	−1.17^†^	−1.26^†^	4.82E-14	0.52	Yes*
SH3GL2	6456_at	13.58	−1.45^†^	−1.20^†^	1.02E-12	0.51	Yes
TUBB6	84617_at	12.74	1.13^†^	1.31^†^	2.69E-21	3.74	No
NRSN1	140767_at	12.64	−1.05^†^	−1.09^†^	1.11E-14	0.51	No
FAM19A2	338811_at	12.51	−1.22^†^	−1.48^†^	3.38E-16	0.44	No
NALCN	259232_at	11.23	−1.00^†^	−1.08^†^	1.74E-15	0.49	No
CDH18	1016_at	10.67	−1.10^†^	−1.09^†^	1.14E-13	0.52	No
GNAL	2774_at	10.43	−1.05^†^	−1.11^†^	8.82E-12	0.51	No
FN1	2335_at	9.33	1.50^†^	1.18^†^	1.46E-12	2.37	Yes*
COL1A2	1278_at	8.83	1.62^†^	1.52^†^	1.03E-17	3.48	Yes*
COL4A2	1284_at	7.89	1.81^†^	1.72^†^	1.46E-20	3.43	Yes*
COL1A1	1277_at	7.75	2.12^†^	2.34^†^	1.76E-17	3.25	Yes
COL3A1	1281_at	7.61	2.29^†^	2.11^†^	7.00E-18	3.09	Yes*
COL4A1	1282_at	7.52	2.06^†^	1.84^†^	1.62E-19	3.43	Yes*

**Figure 6:**
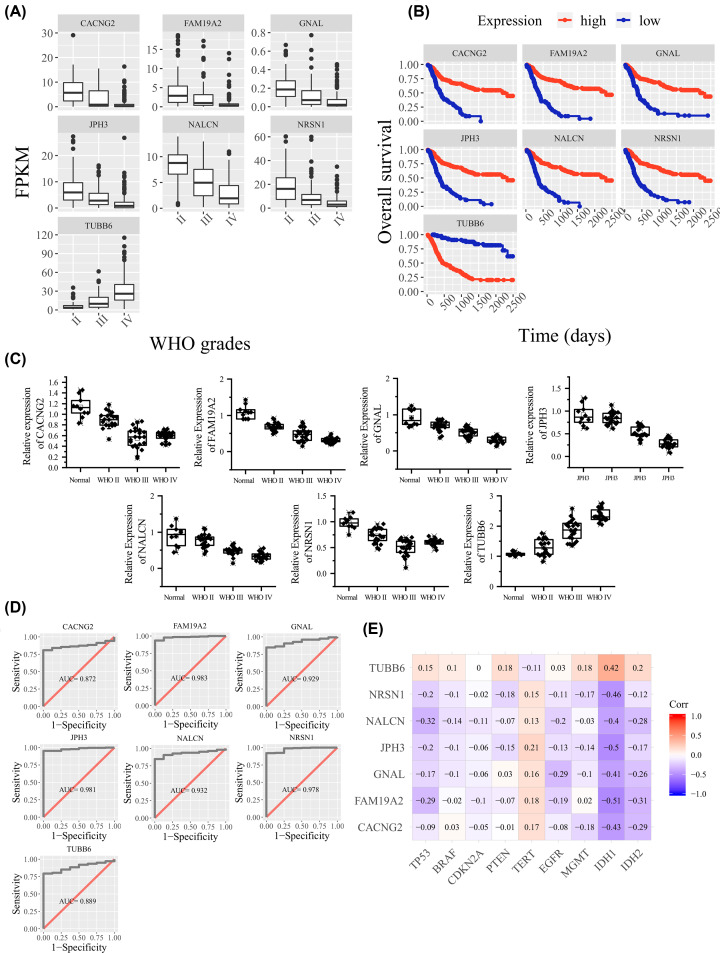
Expression of unreported genes and their clinical significance (**A**) Boxplot for genes are not reported in glioma using CGGA mRNAseq FPKM data. (**B**) Survival plot for genes which was not reported in glioma using CGGA mRNAseq FPKM data. (**C**) Boxplot based on the relative expression of each gene, as detected by qPCR. (**D**) ROC curve for unreported genes in glioma using GSE16011. (**E**) A graphical display of a correlation matrix using data from GSE16011.

Connectivity*, connectivity of nodes (genes) in network (Figure 6C); logFC G3vsG2* indicates log2 fold change of DEGs (WHO III vs WHO II); logFC G4vsG3* indicates log2 fold change of DEGs (WHO IV vs WHO III); *Likelihood test P-*val^*^ indicates *P*-value obtained through survival analysis. Yes^*^ is just reported through data analysis, without experiment conducted to verify functions of genes in glioma. RR^*^, relative risk of death was obtained through computing method described in the method.

† Indicates that *P*-value of DEG is less than 0.001.

Twenty-four key genes were identified in the present study. Most of them were reported in glioma, CACNG2 JPH3, TUBB6, NRSN1, FAM19A2, NALCN, GNAL have not been reported yet. Based on our current results, the researchers believe that some of these genes may have important research implications for gliomas. The results indicated that the expression of CACNG2, JPH3, NRSN1, FAM19A2, NALCN, GNAL decreased as the WHO grade increased. Their higher expression levels indicate a better prognosis (Figure 6A,B). TUBB6 increased as the WHO grade increased, higher expression levels of TUBB6 indicates a poor prognosis (Figure 6A,B). We used qRT-PCR to detect differences in the expression of these genes. The results show that the differences in these genes exist objectively (Figure 6C). ROC analysis showed that the expression levels of these genes have a good diagnostic efficacy on gliomas (Figure 6D).

### Co-expression of unreported key genes and known glioma biomarker

We selected nine markers associated with gliomas, TP53, BRAF, CDKN2A, PTEN, TERT, EGFR, MGMT, IDH1, IDH2. Pearson correlation analysis was performed on them. There was not a strong correlation between unreported key genes and known glioma biomarker (Figure 6E). But IDH1 have a significant correlation with each unreported key gene (*P*<0.05).

## Discussion

Glioma has become an important factor threatening human life. Research on glioma pathogenesis is important. As an exploratory research, the study aims to to discover key genes related to tumor malignancy. Paving the way for the next project is our lab. Therefore, datasets GSE16011 and CGGA with larger sample size were first selected so as to carry out WGCNA, differential analysis and survival analysis. The most representative top 20% genes in WGCNA module significantly related to WHO grades were selected. KEGG pathway analysis suggests that these genes are enriched with Cell cycle, ECM–receptor interaction, p53 signaling pathway, DNA replication, Focal adhesion, Small cell lung cancer and PI3K-Akt signaling pathway related to tumor functions (Table 2) while GO enrichment analysis shows that they are enriched with platelet-derived growth factor binding, extracellular matrix structural constituent, structural molecule activity, phospholipase A2 inhibitor activity and protein binding pathway (Table 3). Genes mainly involved in GO enrichment analysis include ANXA1, COL1A1, COL1A2, COL3A1, COL4A1, COL4A2, TUBB6, ANXA1 and ANXA2. It is obviously seen that various subtypes of collagen play important roles in glioma. COL1A1 has been researched in glioma.

**Table 2:** KEGG pathway enrichment analysis for 24 genes

Pathway description	Observed gene count	False discovery rate	Matching proteins
Amoebiasis	7	6.62E-09	COL1A1, COL1A2, COL3A1, COL4A1, COL4A2, FN1, GNAL
ECM–receptor interaction	6	7.82E-08	COL1A1, COL1A2, COL3A1, COL4A1, COL4A2, FN1
Protein digestion and absorption	6	3.96E-06	COL1A1, COL1A2, COL3A1, COL4A1, COL4A2
Focal adhesion	6	7.17E-06	COL1A1, COL1A2, COL3A1, COL4A1, COL4A2, FN1
PI3K-Akt signaling pathway	6	1.13E-04	COL1A1, COL1A2, COL3A1, COL4A1, COL4A2, FN1
Small cell lung cancer	3	6.08E-03	COL4A1, COL4A2, FN1
Platelet activation	3	1.72E-02	COL1A1, COL1A2, COL3A1

**Table 3:** GO enrichment analysis for 24 genes

Pathway description	Observed gene count	False discovery rate	Matching proteins
Platelet-derived growth factor binding	4	1.97E-06	COL1A1, COL1A2, COL3A1, COL4A1
Extracellular matrix structural constituent	5	2.13E-05	COL1A1, COL1A2, COL3A1, COL4A1, COL4A2
Structural molecule activity	7	3.08E-03	ANXA1, COL1A1, COL1A2, COL3A1, COL4A1, COL4A2, TUBB6
Phospholipase A2 inhibitor activity	2	6.50E-03	ANXA1, ANXA2
Protein binding	14	4.70E-02	ANXA1, ANXA2, COL1A1, COL1A2, COL3A1, COL4A1, FN1, GNAL, KCNB1, KIF20A, SERPINH1, SH3GL2, TIMP1, VIM

Twenty-four key genes were identified in the study. TOM matrix generated in WGCNA was used to build co-expressed network for 24 genes. Differential expression analysis was just made for GSE16011 and CGGA mRNA seq FPKM data in data analysis process. It is shown in Figure 5A,B that 24 key genes have obvious differential expression in mRNA microarray data, which is consistent with previous conclusion, proving that differential expression of 24 genes is of higher stability and credibility.

A total of 2682 genes identified in survival analysis study (shown in Supplementary ResultC.csv) are simultaneously expressed to be significantly related to prognoses of patients in two CGGA datasets, which is of great reference significance for other glioma studies. All 24 key genes identified in previous study belong to result C. Functions of KIF20A, CLIC1, ANXA2, ANXA1, TIMP1, VIM, TACC3, SH3GL2, COL1A1, CDH1,8 [14] SERPINH1/HSP47 [15] were reported via experimentation in glioma. KCNB1, FN1, COL1A2, COL4A2, COL3A1, COL4A1 was just reported by data analysis; however, functional experiments of those genes were not conducted. In addition, CACNG2, JPH3, TUBB6, NRSN1, FAM19A2, NALCN, GNAL were not reported in glioma. Researched and reported genes prove that conclusion of the study is of higher credibility. The results of ROC analysis with areas under the curve ranging from 0.85 to 0.99, reflecting a fair to excellent diagnostic efficiency of these unreported proteins.

To investigate the relationship between the unreported genes we obtained the important clinical markers. We selected nine markers associated with gliomas, TP53, BRAF, CDKN2A, PTEN, TERT, EGFR, MGMT, IDH1, IDH2. Pearson correlation analysis was performed on them. The results showed that these genes were significantly associated with IDH1 (Figure 6B).

We checked if these key genes have been reported by searching in PubMed. Results of searching in PubMed, with the summary of survival analysis and differential expression analysis of key genes is presented in Table 1. TUBB6 has higher RR in unreported key genes. Official full name of TUBB6 is tubulin β 6 class V, belonging to tubulins family. As a spherical molecule, Tubulin, divided into α-tubulin and β-tubulin, serves as the basic molecule constituting microtubule. As a part of cytoskeleton, microtubule is spread all over the whole cytoplasm. It is reported that subtype mutation of tubulin is involved in abnormalities of brain [16]. Participating in maintaining structure of cell, constituting cytoskeleton with microfilament and intermediate filament, microtubules plays a is very important role in many biological processes. They are also involved in constituting cilium and flagellin, which are applied to intracellular transport and participate in several cellular processes, including motion of secretory vesicles, organelle and substances in the cell. Moreover, they participate in cell division (mitosis and meiosis), for example, to form spindle apparatus used to pull eukaryotic chromosome.

There are lots of reports about key roles of microtubule in tumor such as lymphoblastic leukemia cells [17], glioma [18]. In this research, we found that TUBB6 is significantly related to glioma grades and poor prognosis, indicating that TUBB6 may play an important role in glioma genesis and development process.

## Conclusions

Twenty-four key genes were hunted from hub genes enriched with key pathway relating to tumor of modules of WGCNA. Twenty-four key genes were potential prognostic markers for glioma. KIF20A, CLIC1, ANXA2, ANXA1, TIMP1, VIM, TACC3, SH3GL2, COL1A1, SERPINH1/HSP47, CDH18 have been reported in glioma, along with carrying out functional experiment. As to reports about KCNB1, FN1, COL1A2, COL4A2, COL3A1, COL4A1, only data analysis has been done; however, functional experiments were not conducted. CACNG2, JPH3, TUBB6, NRSN1, FAM19A2, NALCN, GNAL have not been reported in gliomas. They are novel biomarkers and independent prognostic factors in gliomas, but require experiments at the protein level.

## Data Availability

The datasets analyzed during the current study are available in the CGGA repository [data3, data4 http://www.cgga.org.cn/download.jsp]. The datasets analyzed during the current study are available in the GEO repository [https://www.ncbi.nlm.nih.gov/geo/query/acc.cgi?acc=GSE16011].

## Abbreviations

ANXA1: annexin A1
ANXA2: annexin A2
CACNG2: calcium voltage-gated channel auxiliary subunit gamma 2
CDH18: cadherin 18
CGGA: Chinese Glioma Genome Atlas
circRNA: circular RNA
CLIC1: chloride intracellular channel 1
COL1A1: collagen type I alpha 1 chain
COL1A2: collagen type I alpha 2 chain
COL3A1: collagen type III alpha 1 chain
COL4A1: collagen type IV alpha 1 chain
COL4A2: collagen type IV alpha 2 chain
DEG: differential expressed gene
FAM19A2: family with sequence similarity 19 member A2
FN1: fibronectin 1
FPKM: Fragments per Kilobase Million
GEO: Gene Expression Omnibus
GNAL: G protein subunit alpha L
GO: Gene Ontology
JPH3: junctophilin 3
KCNB1: potassium voltage-gated channel subfamily B member 1
KEGG: Kyoto Encyclopedia of Genes and Genomes
KIF20A: kinesin family member 20A
lncRNA: long non-coding RNA
ME: module eigenvector
miRNA: MicroRNA
MM vs GS: Module Membership vs Gene significance
mRNA: messenger RNA
NALCN: sodium leak channel, non-selective
NRSN1: neurensin 1
qRT-PCR: Quantitative Real-time Polymerase Chain Reaction
ROC curve: receiver operating characteristic curve
SERPINH1: serpin family H member 1
SH3GL2: SH3 domain containing GRB2 like 2, endophilin A1
TACC3: transforming acidic coiled-coil containing protein 3
TIMP1: TIMP metallopeptidase inhibitor 1
TOM: topological overlap matrix
TUBB6: tubulin β 6 class V
VIM: vimentin
WGCNA: weighted correlation/co-expression network analysis
WHO: World Health Organization
